# Hyperresponsiveness to inflammatory stimulation in human neuronal-like cells from patients with inflammation-associated depression

**DOI:** 10.3389/fpsyt.2025.1576880

**Published:** 2025-10-29

**Authors:** Susanne Elisa Schillo, Carmen Schiweck, Mareike Aichholzer, Anna Y. Yotova, Tsendmaa Tsengenbayar, Sharmili Edwin Thanarajah, Andreas Reif, Florian Freudenberg

**Affiliations:** ^1^ Department of Psychiatry, Psychosomatic Medicine and Psychotherapy, Goethe University Frankfurt, University Hospital, Frankfurt, Germany; ^2^ Faculty of Biological Sciences, Goethe University Frankfurt, Frankfurt, Germany; ^3^ Fraunhofer Institute for Translational Medicine and Pharmacology ITMP, Frankfurt am Main, Germany

**Keywords:** depression, inflammation, *in vitro* model, neurodevelopment, neuroinflammation, stem cells

## Abstract

**Introduction:**

Chronic low-grade inflammation has been associated with the development and progression of depression. However, its impact on neuronal development and connectivity remains poorly understood. In this study, we investigated how chronic and acute inflammation may influence early neurite development in neuronal-like cells (monocyte-derived neuronal-like cells, MDNCs).

**Methods:**

Circulating monocytes were obtained from healthy controls (HC, n = 5), patients with major depression (MD, n = 4), patients with depression and elevated peripheral inflammatory markers (MD-INF, n = 4) and patients with schizophrenia (SZ, n = 5). Monocytes were transdifferentiated into MDNCs and either left untreated (CTRL) or exposed to lipopolysaccharide (LPS, 100 ng/mL) to induce an acute inflammatory response. Neurite morphology was quantified using MAP2 immunofluorescence staining and microscopic analysis.

**Results:**

Transdifferentiation rates were significantly higher in MD-INF cultures compared with HC, MD and SZ. Sholl analysis demonstrated that MD-INF MDNCs treated with LPS exhibited a significantly greater neurite ending radius than MD and SZ cells or MD-INF under control conditions. Similarly, MD-INF cells treated with LPS showed more Sholl intersections compared with SZ cells treated with LPS and MD-INF cells under control conditions. Furthermore, the average neurite length of MD-INF cells treated with LPS exceeded that of MD and SZ cells treated with LPS and MD-INF cells under control conditions.

**Discussion:**

These findings suggest a hyperresponsive phenotype to inflammatory stimulation *in vitro* in early neurons from patients with depression and elevated peripheral inflammation. Our results may provide novel insights into how inflammation could influence neurodevelopmental processes relevant to depression and may serve as a model for further investigations regarding targeted interventions.

## 1 Introduction

Depression remains one of the leading causes of disability, underscoring the persistent challenge in achieving effective treatment (1, 2). Approximately one-third of patients fail to respond to conventional antidepressants that target monoaminergic systems (3). Escalated treatment strategies such as antipsychotics, lithium, electroconvulsive therapy and, more recently, esketamine (4), can only partially address this treatment gap but are insufficient for many patients. A deeper understanding of the neurobiological underpinnings of depression and improved patient stratification are essential for developing faster and more individualized treatment strategies.

A growing body of evidence supports a link between inflammation and depression (5) in a subset of patients (6). Interestingly, elevated peripheral inflammatory markers have been associated with difficulties in treating depression (7). Peripheral inflammation has been proposed to contribute to neuroinflammation, potentially through mechanisms such as increased blood-brain-barrier permeability (8). In the brain, inflammation seems to decelerate dendritic growth in neurons of the hippocampus (9). In a rodent model, chronic exposure to bacterial lipopolysaccharides (LPS), mimicking chronic inflammation, reduces dendritic length in immature newborn granule cells in the hippocampus (10). Human neuroimaging studies similarly report reduced hippocampal volume in patients with depression (11). However, direct insights into how inflammation affects human neurons in patients with depression are sparse, largely due to the difficulty of accessing viable patient-derived neuronal tissue. Like depression, schizophrenia has been associated with immune dysregulation (12), but the molecular mechanisms linking inflammation to disease pathology are not yet fully understood.

To investigate these questions, various cell models have been used to study neuronal pathologies in psychiatric disorders. Human induced pluripotent stem cells (hiPSC) represent one of the most significant systems (13). Despite their overwhelming potential, the reprogramming required for hiPSC production can elicit epigenetic alterations that may confound the results (14). Additionally, generating hiPSC can be time-consuming and concerns regarding reproducibility have been raised (15). In 2018, Bellon et al. (16) introduced a new approach using human circulating monocytes, which possess pluripotent capacities, to generate monocyte-derived neuronal-like cells (MDNCs). This technique requires 20 days and does not require reprogramming (16), which enables the study of early neuronal development from living human donors efficiently while avoiding potential confounders associated with hiPSC reprogramming.

Although depression most often manifests in adulthood, the vulnerability-stress model (17) posits that its pathogenesis begins much earlier, shaped by genetic and epigenetic vulnerabilities. MDNCs, as a model for neurons early in development, carrying the genetic load of the donor, represent a promising model to study potential neurodevelopmental pathologies in depression.

Here, we generated MDNCs from patients with depression (with [MD-INF] and without [MD] increased levels of inflammatory markers), patients with schizophrenia (SZ), and healthy controls (HC). Our objectives were to examine group differences in monocyte transdifferentiation rates, dendritic growth, and response to acute inflammatory stimulation. We hypothesized that SZ-derived MDNCs would exhibit the lowest differentiation efficiency and neurite growth, followed by MD-INF, MD and HC MDNCs, respectively. To our knowledge, this is the first study to use MDNCs from patients with depression and to assess their response to inflammatory stimulation.

## 2 Materials and methods

### 2.1 Participants

**Figure 1:**
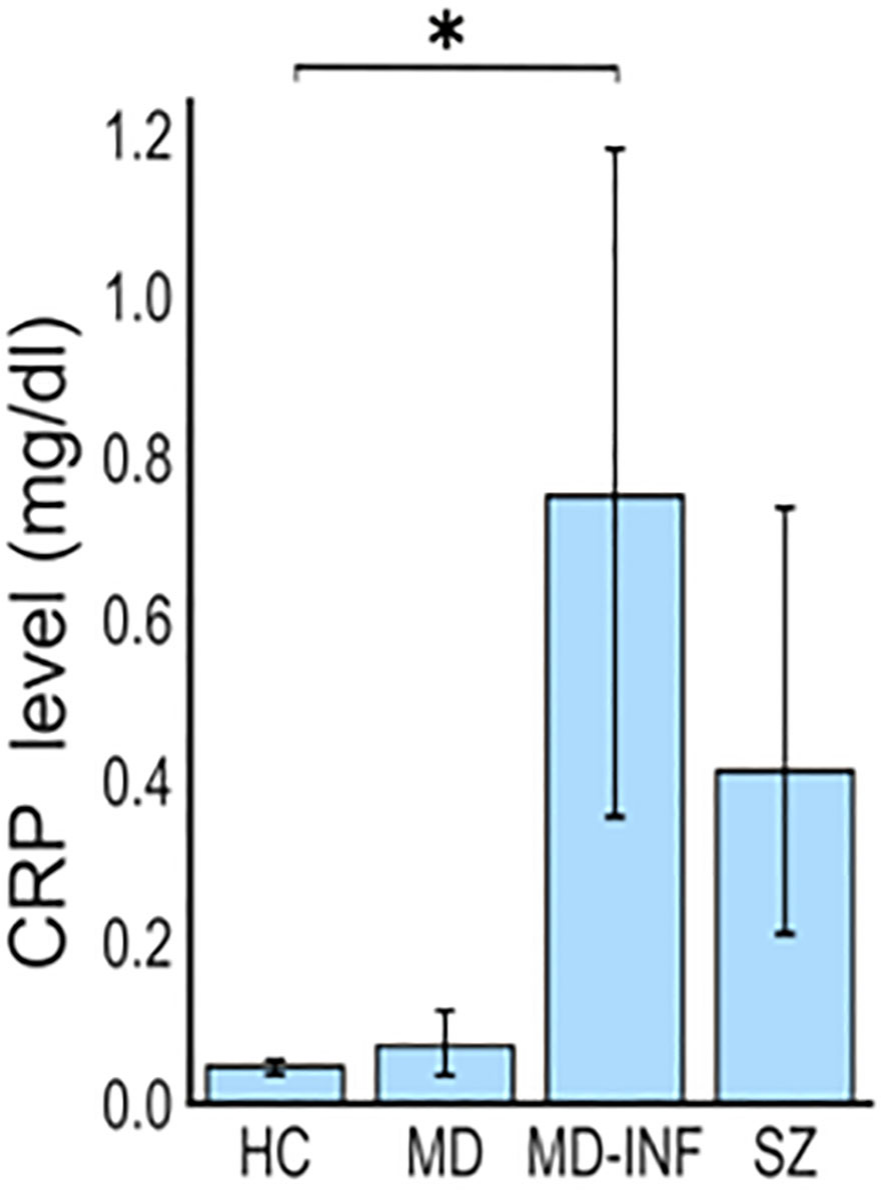
Elevated CRP levels in patients with depression and inflammation (MD-INF) compared to patients with depression without inflammation (MD), healthy controls (HC) and patients with schizophrenia (SZ). Patients were included based on CRP blood levels, which were significantly elevated in MD-INF subjects (CRP: 0.75 mg/dl (± 0.46)) compared to HC (CRP: 0.05 mg/dl (± 0.01)). Data are displayed as mean ± standard deviation. For statistical analysis Kruskal-Wallis test was used followed by Dunn’s *post-hoc* test with Holm correction. Statistically significant differences are indicated by asterisks: *p<0.05.

The sample cohort consisted of 18 adult Caucasian participants: 5 HC, 4 MD patients, diagnosed according to International Classification of Diseases 10^th^ Revision (ICD-10) criteria (F32.2/F33.2), without elevated inflammatory markers, 4 MD patients with consistently elevated blood levels of CRP, representing a chronic inflammatory status (i.e. MD-INF), and 5 patients with schizophrenia (ICD-10 F20.0; i.e. SZ; for cohort demographics see **Table 1**). During the recruitment period, every patient admitted to the hospital was screened for symptoms of depression and elevated C-reactive protein (CRP) levels. For the MD-INF group, CRP levels in the blood were measured at least five different time points over a period of four months or more. CRP levels on the day of the blood draw were significantly higher compared to the HC group (**Table 1**, **Figure 1**). These measurements prior to admission were due to previous inpatient or outpatient visits, during which CRP is routinely assessed. HC subjects were recruited from the University Hospital Frankfurt staff and their relatives and were screened for mental illness using the Mini-International Neuropsychiatric Interview (M.I.N.I.) diagnostic interview for psychiatric disorders (18) before inclusion. The recruited patients were in inpatient treatment for the respective disorder at the Department of Psychiatry, Psychosomatic Medicine and Psychotherapy of the University Hospital Frankfurt, and were diagnosed by experienced clinicians.

**Table 1:** Age and BMI for the study sample.

	HC	MD	MD-INF	SZ	Statistics
Sample size (F/M)	5 (4/1)	4 (4/0)	4 (0/4)	5 (0/5)	
Age in years (SD)	33.4 (10.78)	41.25 (11.5)	42.0 (16.02)	28.4 (4.83)	F3,14 = 1.56, p = 0.244
BMI in kg/m^2^ (SD)	22.54 (2.34)	24.03 (2.25)	29.18 (7.16)	24.32 (4.41)	H(3) = 4.89, p = 0.18
CRP in mg/dl (SD)	0.046 (0.011)	0.063 (0.032)	0.753 (0.455)	0.412 (0.353)	H(3) = 13.16, p = 0.004

BMI, body mass index; F, female; HC, healthy controls; CRP, C-reactive protein, M, male; MD, patients with major depression; MD-INF, patients with major depression and elevated inflammatory markers; SZ, patients with schizophrenia, data is presented as mean (SD).

As this was a naturalistic, clinical sample, the patients were treated with different psychopharmacological medication. In the MD group, the following antidepressants were administered: venlafaxine, sertraline and escitalopram. Additionally, lithium and quetiapine were used in some of the patients as augmentation strategies. In the MD-INF group, sertraline, venlafaxine and bupropion were used, along with lithium in one patient. For the SZ group, medication included paliperidone, clozapine, olanzapine and cariprazine. One patient in this group also received venlafaxine but was in remission from depressive symptoms (total score BDI-II: 11/63 equivalent to a “mild mood disturbance”).

Exclusion criteria for both the HC and patient groups were clinical symptoms of an acute infectious disease, known chronic infectious diseases such as HIV or hepatitis, severe neurological diseases, and severe somatic diseases (for example severe heart failure or a recent cancer diagnosis). Since blood samples were taken within two weeks after COVID vaccination in two SZ subjects, they were excluded from the study. In the control group, three participants had to be excluded due to insufficient cell adhesion. One participant in the MD-INF group was excluded because CRP levels were within the normal range on the day of sampling. One participant in the MD-INF group had a diagnosed autoimmune disorder (psoriatic arthritis, see **Table 2**). However, exploratory analyses excluding this individual revealed similar results; therefore, the participant was retained in the study. We informed both HC and patient groups orally and in writing about the study and they gave written informed consent to participate. The study was approved as part of the genome-wide analysis of the genotype-phenotype relationship in the long-term course of disorders from the affective and psychotic spectrum (Approval ID 425/14) by the Ethics Committee of the Faculty of Medicine of the Goethe University Frankfurt am Main on December 16^th^, 2016, and complied with the Declaration of Helsinki (version 63rd, 2008).

**Table 2:** Somatic conditions and medication of patients as documented in the medical report.

Patient #	Somatic conditions	Somatic medication
MD-01	Polycystic Ovary Syndrome	Progesterone 200 mg/day; Paracetamol as needed: 1 g two days before blood collection
MD-03	Thyroidectomy; Prolactinoma	Cabergoline 0.5 mg per week; L-thyroxine 125 µg daily; Metoprolol 47.5 mg twice daily for restlessness; Macrogol (also known as Polyethylene Glycol (PEG) once daily
MD-04	Hypercholesterolemia; disc prolapse; bursitis of the glenohumeral joint; peritonitis with adnexitis (~11 years before blood collection); adnexectomy and oophorectomy; ovarian cystectomy (~25 years before blood collection)	Ibuprofen as needed: 600 mg 6 and 7 days before blood collection probably due to pain in the shoulder
MD-05	Graves’ disease (Basedow’s disease) - no longer requires substitution; status post traumatic brain injury; status post AV reentry tachycardia (ablation); vitamin D deficiency	Atorvastatin 20 mg daily; Vitamin D 20,000 IU per week
MD-INF-01	Astigmatism; status post laser surgery	Thealoz Duo (Trehalose, Hyaluronic acid) eye drops 1-0-1; Corneregel (Dexpanthenol) eye drops 0-0-1
MD-INF-02	Heartburn	Pantoprazole 20 mg once daily
MD-INF-03	No known conditions at the time of hospital admission	Ibuprofen 400 mg as needed: taken several times probably due to headache
MD-INF-04	Psoriatic arthritis, arterial hypertension; Vitamin D deficiency	Folic acid + Vitamin B12 + Vitamin B6; Bisoprolol; Leflunomide; Pantoprazole; Vitamin D3
SZ-01	Vitamin D deficiency	Vitamin D 20,000 IU per week
SZ-02	Bronchial asthma	Propranolol 70mg/day; Ramipril 5mg/day
SZ-03	No known conditions at the time of hospital admission	Ibuprofen and Metamizole as needed (6 days before blood collection: Ibuprofen 400 mg and Metamizole 500 mg; from 5 days before until blood collection: Ibuprofen 400 mg three times daily due to toothache)
SZ-04	Various fractures after a fall the year before blood collection	none
SZ-05	Vitamin D deficiency	Vitamin D 20,000 IU per week; Omega-3 fatty acids (Omacor) once daily; Erythropoietin (EPO) 50,000 IU per week

Medication prescribed on an as-needed basis (PRN) was documented only during the week preceding the blood draw.

### 2.2 Transdifferentiation of circulating monocytes

**Figure 2:**
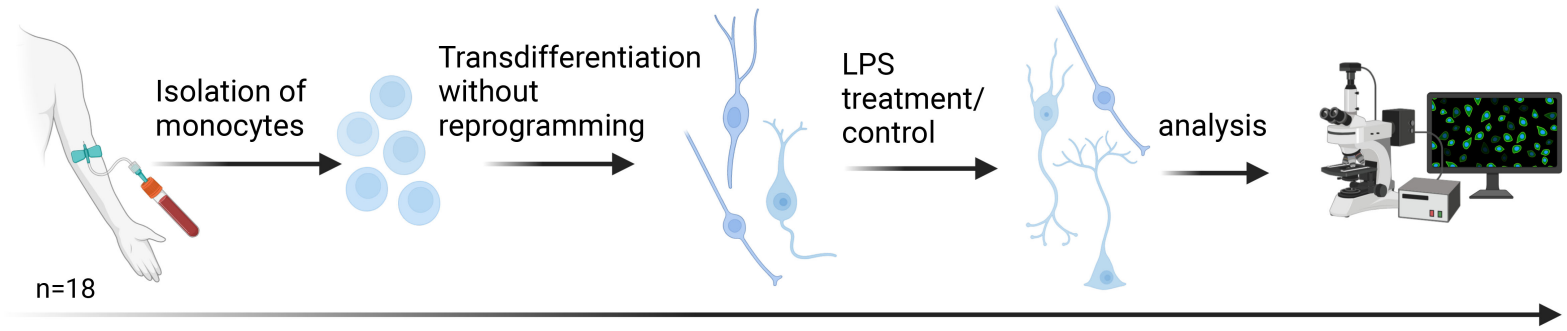
Transdifferentiation of human monocytes into neuronal-like cells. Following the protocol of Bellon et al., monocyte-derived neuronal-like cells (MDNCs) were generated within 20 days without reprogramming. On day 20, cells were exposed to lipopolysaccharide (LPS) for 24 h as a short-term inflammatory stimulus. Samples were obtained from 18 participants: healthy controls (HC, n = 5), patients with major depression (MD, n = 4), patients with depression and elevated peripheral inflammatory markers (MD-INF, n = 4), and patients with schizophrenia (SZ, n = 5). Created with BioRender.com.

For transdifferentiation of circulating monocytes, we followed the procedure described by Bellon et al. (16) and added an inflammatory stimulus (see **Figure 2**). Specifically, we separated peripheral blood mononuclear cells (PBMCs) from 30–40 ml freshly drawn blood using Ficoll-Paque (order #: 17-1440-03, lot: 10038368GE, GE Healthcare). For each individual, we coated one 25 cm² flask (order #: 353109, BD Falcon) with fibronectin (order #: F2006, Sigma-Aldrich) and then cultured 13.5 million PBMCs. From the remaining PBMCs, we isolated CD14+ monocytes by immunomagnetic separation, using the MACS Magnetic separator (Miltenyi Biotec) and human CD14 microbeads (order #: 130-050-201, Miltenyi Biotec). We added human recombinant macrophage colony stimulating factor to the monocytes at a concentration of 1:2,000 (order #: 300-25, Abcys). We added coverslips (order #: 0111520, Marienfeld GmbH & CoKG) in 24-well-plates (order #: 353047, BD Falcon) and coated with fibronectin before cultivating the monocytes at a concentration of 180,000 cells/cm². For culturing, we used Dulbecco’s Modified Eagle Medium (DMEM) (order #: 61965059, ThermoFisher Scientific) with 1% antibiotics (order #: 15240-062, ThermoFisher Scientific) and 10% Fetal Bovine Serum (FBS, order #: 10270-106, lot: 41G8072K, ThermoFisher Scientific). We incubated the cells for four days at 37 °C and 5% CO_2_. The used PBMC medium was supplemented 2:1 with fresh DMEM and added to the monocyte culture. On day seven, we exchanged the used medium with fresh DMEM and added butylated hydroxyanisole (BHA, order #: B1253-100G, Sigma-Aldrich) to a final concentration of 50 nM. We replaced PBMC media with fresh DMEM and mixed the used PBMC medium 1:1 with DMEM before adding it to the monocyte culture. On day ten, we added BHA at a final concentration of 50 nM and Retinoic acid (RA, order #: R2625, Sigma-Aldrich) at a final concentration of 16 µM to the monocytes. We supplemented the medium with PBMC medium as described on day seven. On day 13, we added BHA (final concentration: 50 µM), RA (final concentration: 16 µM), Insulin-like growth factor-1 (IGF-1, order #: 100-11, Peprotech; final concentration: 12.5 ng/ml) and Neurotrophin-3 (NT-3, order #: 450–03 Peprotech; final concentration: 30 ng/ml) to the monocyte culture, and supplemented cultures with PBMC medium as described. On day 17, we added KCl (final concentration: 25 mM). On day 20, we treated cells with LPS (100 ng/ml, order #: L2630, Sigma-Aldrich) or added medium as a control. After 24 hours of treatment (+/-1 hour), we washed the cells with PBS, then fixed them with Histofix containing 4% sucrose (P087, Carl Roth) for 10 min and subsequently washed two times for 5 min with PBS. We collected cell supernatant before and after LPS treatment for use in the Enzyme-linked Immunosorbent Assay (ELISA).

Although experiments were not blinded to group, all cultures were processed using identical protocols, reagents, and time points to minimize procedural variability.

### 2.3 Immunofluorescence

After washing, we permeabilized the cells with 0.1% Triton X-100 in PBS for 10 min, followed by preblocking in 5% BSA in PBS for one hour. Subsequently, we added primary antibodies (Microtubule-associated protein 2, MAP2, RRID: AB_2138181; Synaptic systems) at a concentration of 1:1,000 in 1% BSA in PBS and incubated the plates for 36 hours on a shaker at 4°C. Afterwards, we washed the cells 3 times with PBS for 5 min and treated them with secondary antibodies (goat anti-mouse Alexa Fluor 555 [RRID: AB_2535844], goat anti-guinea pig Alexa Fluor 647 [RRID: AB_2735091], ThermoFisher Scientific) at a dilution of 1:500 in 1% BSA in PBS for 1 hour at room temperature. After washing with PBS three times for 5 min, we mounted the cell-containing coverslips with Fluoroshield containing 4’,6-Diamidino-2-phenylindole (DAPI; order #: F6057, Sigma-Aldrich).

### 2.4 Microscopy and tracing

We captured pictures using a Zeiss AxioObserver.Z1 microscope equipped with an Objective A-Plan 10x/0.25 Ph1 M27. We used six coverslips without LPS treatment for each subject and three coverslips with LPS treatment. To ensure a comprehensive yet unbiased analysis of the cultured cells, we randomly selected five regions per coverslip and captured images for evaluation. Neuronal-like cells were selected for tracing if they met the following criteria: at least three primary neurites, each < 5 µm in diameter and > 12 µm in length, and a soma diameter < 50 µm. These criteria ensured that only transdifferentiated cells were included in the analysis. We performed semi-automated tracing using the SNT plugin (19) running in the Fiji/ImageJ distribution (https://fiji.sc/) (20). In the SNT plugin we used Sholl analysis (21) to describe neurite arborization of MDNCs. In total, we performed Sholl analysis on 1,167 cells. Specifically, for HC, we analyzed 105 cells in the CTRL group and 64 in the LPS-treated group. For MD, 127 cells were analyzed in the CTRL group and 85 in the LPS-treated group. In the MD-INF group, we examined 326 cells in the CTRL condition and 213 in the LPS-treated condition. Lastly, for SZ, 154 cells were analyzed in the CTRL group and 93 in the LPS-treated group.

### 2.5 ELISA for interleukin 6

To measure interleukin 6 (IL-6) levels we employed the ELISA MAX™ Deluxe Set Human IL-6 (cat. # 430504, Biolegend) following the manufacturer’s instructions. In brief, we added 100 µl of Capture Antibody solution to all wells of a 96-well plate, sealed the plate and incubated overnight at 4°C. After that, we washed the plate, followed by blocking with 200 µl of 1X Assay Diluent for 1 h on a plate shaker at room temperature. After washing, we added 100 µl of standard or samples to the plate and incubated for 2 h at room temperature on a shaker. We washed again and after adding 100 µl of Detection Antibody, incubated at room temperature for 1 h shaking. We washed again and incubated with 100 µl of avidin-horseradish peroxidase for 30 min at room temperature on the shaker. After washing, we added 100 µl of substrate solution for 15 min in the dark. Finally, we added stop solution and measured the absorbance at 450 nm using a microplate reader (Infinite M200 PRO by Tecan Austria GmbH, Salzburg, Austria). We calculated the IL-6 concentration in the samples with the calibration curve in pg/ml.

### 2.6 Statistical analysis

Demographic characteristics (Age) followed a normal distribution both after visual inspection and according to the Shapiro-Wilk test (p>0.05) and was compared using one-way analysis of variance (ANOVA). Since transdifferentiation rates also followed a normal distribution, we applied a two-way ANOVA to analyze group (HC, MD, MD-INF, SZ) as between-subject factor and treatment differences (LPS, CTRL) as within-subject factor. Follow-up *post-hoc* comparisons were performed using Tukey’s t-test, controlling for family-wise error rate. Effect sizes are reported as η² for ANOVAs. For CRP, BMI, IL-6 ELISA results, neurite length and Sholl analysis which did not follow a normal distribution (Shapiro-Wilk test p ≤ 0.05), we applied the Kruskal-Wallis test, with group and treatment effects assessed separately, and their interaction analyzed via the combined group x treatment term followed by Dunn’s *post-hoc* test with Holm correction. Effect sizes are reported as ϵ², which represents a more appropriate and interpretable measure for non-parametric tests. A significance threshold of p ≤ 0.05 was applied for all tests, and for ANOVAs Type III sums of squares was used. All statistical analyses were conducted using JASP (v0.19.3).

## 3 Results

### 3.1 Elevated transdifferentiation rates of MDNCs from MD patients with elevated inflammatory markers

Inflammation has been implicated in psychiatric disorders, and elevated levels of inflammation have been observed in a subgroup of MD patients. To assess how elevated exposure to inflammation impacts early neurons, we used the MDNC model developed by Bellon et al. (16).

We recruited healthy controls (HC, n=5), depressive patients *without* elevated inflammatory markers (MD, n=4), depressive patients *with* elevated inflammatory markers (MD-INF, n=4) as well as patients with schizophrenia (SZ, n=5). There were no significant differences in age or body mass index (p>0.05; see **Table 1** for demographic information).

**Figure 3:**
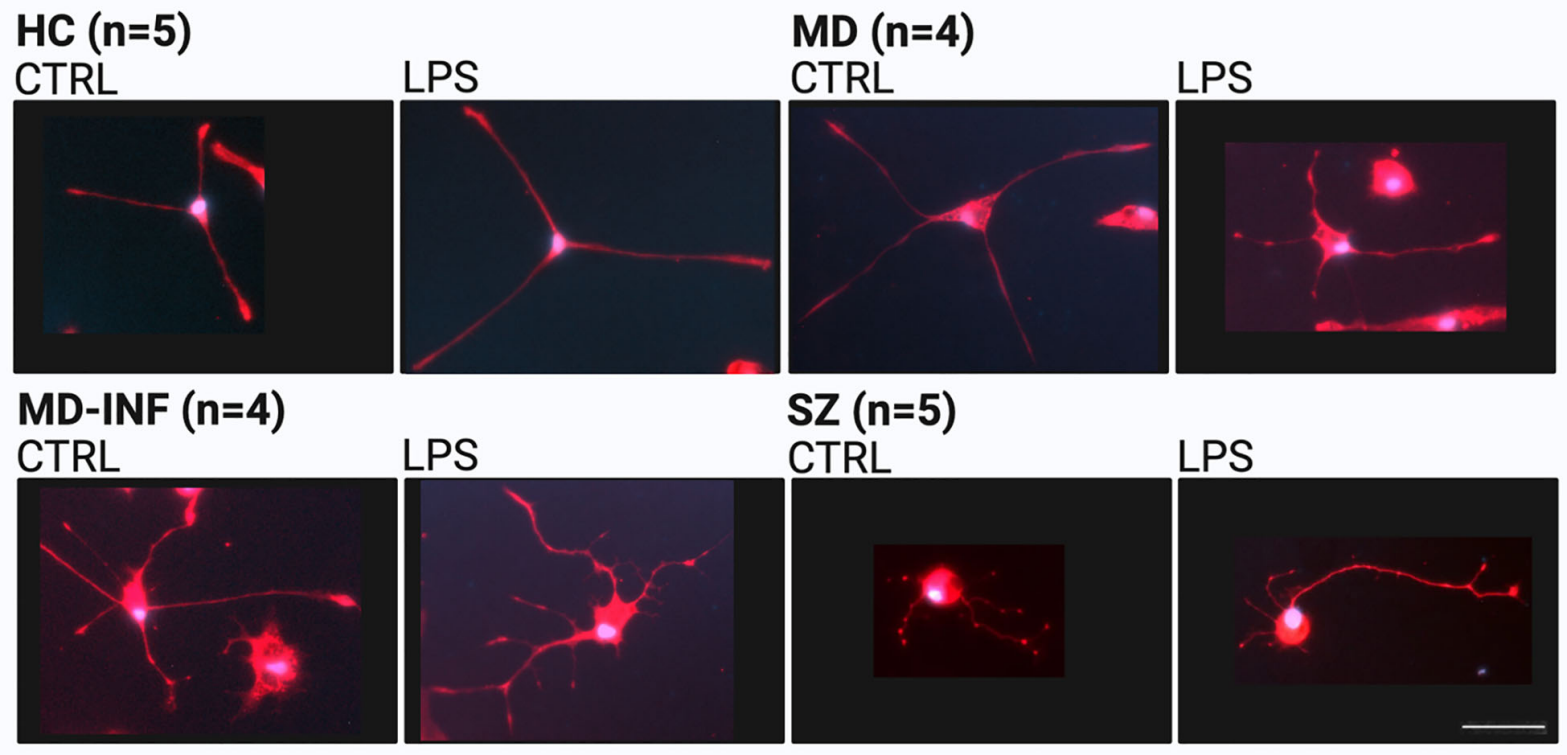
Representative morphology of monocyte-derived neuronal-like cells (MDNCs) stained for microtubule-associated protein 2 (MAP2, red) and 4′,6-diamidino-2-phenylindole (DAPI, blue). Scale bar = 50 µm. HC, healthy controls; MD, major depression; MD-INF, major depression with elevated inflammatory markers; SZ, schizophrenia.

**Figure 4:**
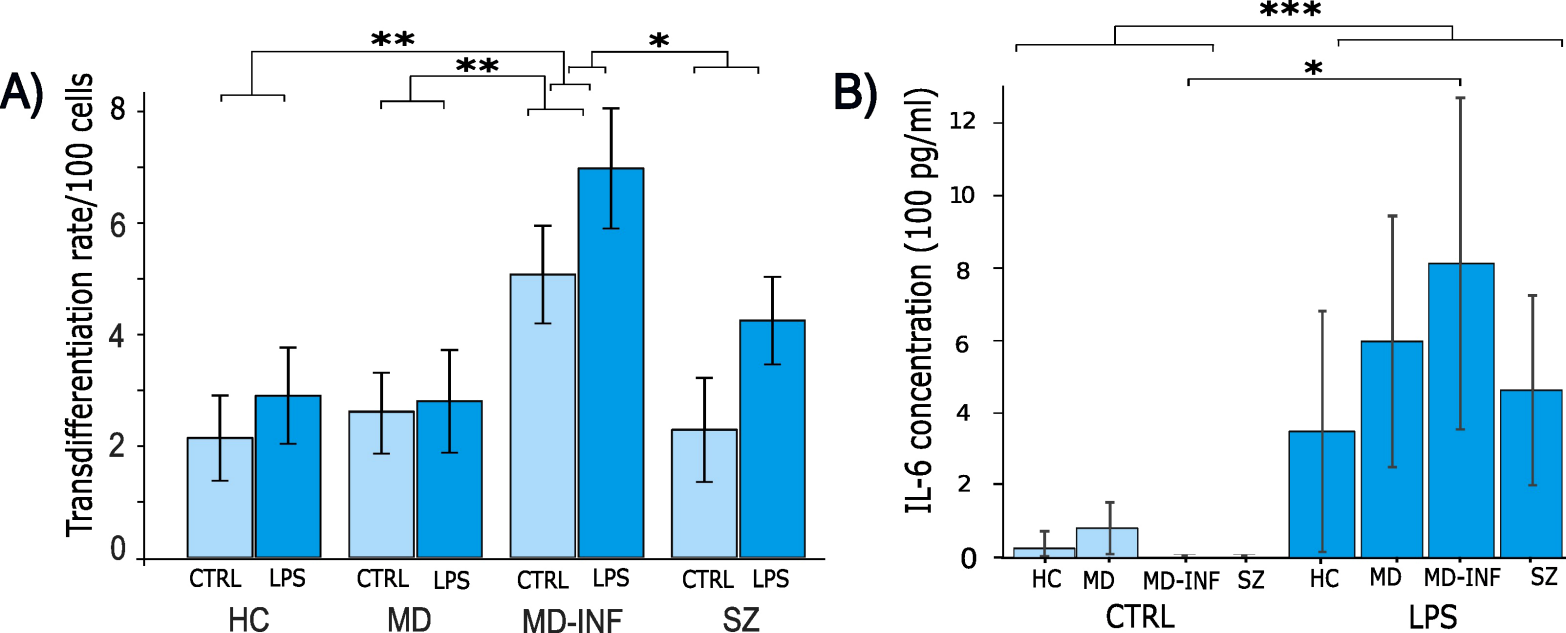
Transdifferentiation rates and IL-6 concentration in supernatants of monocyte derived neuron-like cells. Following transdifferentiation, MD-INF cells exhibited significantly higher transdifferentiation rates from monocytes to MDNCs (CTRL: 5.07 (± 1.75), LPS: 6.98 (± 2.15)) compared to HC (CTRL: 2.25 (± 1.8), LPS: 2.9 (± 1.93)), MD (CTRL: 2.69 (± 1.44), LPS: 2.74 (± 2.38)) and SZ (CTRL: 2.29 (± 2.09), LPS: 4.25 (± 1.76)) **(A)**. IL-6 concentration was measured by ELISA before and after LPS stimulation. The results revealed a substantial increase in IL-6 levels in pg/ml, particularly in MD-INF cells (CTRL: 0.82 (± 1.64), LPS: 812.58 (± 456.2)) compared to HC (CTRL: 26.46 (± 33.06), LPS: 349.17 (± 330.54)), MD (CTRL: 84.87 (± 78.31), LPS: 632.23 (± 273.15)) and SZ (CTRL: 0.0 (± 0.0), LPS: 462.92 (± 260.49)) **(B)**. Abbreviations: CTRL, control; LPS, lipopolysaccharide; HC, healthy control; MD, patients with major depression; MD-INF, MD patients with elevated inflammation, SZ, schizophrenia patients. Data are displayed as mean ± standard deviation. For transdifferentiation rates **(A)**, statistical analyses were performed using two-way ANOVA followed by Tukey’s *post hoc* test. For IL-6 concentrations **(B)**, the Kruskal-Wallis test was applied, followed by Dunn’s *post hoc* test with Holm correction. Statistically significant differences are indicated by asterisks: *p < 0.05, **p < 0.01, ***p < 0.001.

CRP (H(3)=13.16, p=0.004, ϵ²=0.774) was significantly affected (**Figure 1**), with *post-hoc* analysis showing significantly higher CRP in the MD-INF group compared to HC (p_holm_=0.022) and a non-significant difference but trend toward lower value in MD (p_holm_ =0.053). Compared to SZ patients, no significant increase in CRP was observed (p_holm_=1.0) in MD-INF patients. We isolated PBMCs from the blood, followed by the isolation and cultivation of monocytes, which we transdifferentiated into MDNCs. On day 20, we treated cells with LPS (100 ng/ml) or vehicle for 24h (+/-1 hour). After fixation and staining (**Figure 3**), we conducted morphological analysis and determined transdifferentiation rates by counting all cells and transdifferentiated cells. Transdifferentiation rates were defined as the number of transdifferentiated cells per 100 total cells (**Figure 4A**). Transdifferentiated neuronal-like cells were selected based on specific criteria: at least three primary neurites, each < 5 µm in diameter and > 12 µm in length, and a soma diameter < 50 µm. The criteria were based on those established by Bellon et al. (16), with more stringent selection parameters to ensure that only transdifferentiated cells were included. LPS treatment led to a moderate, but statistically non-significant, increase in transdifferentiation rates compared to the control treatment (F_1,28_ = 3.11, p=0.089, η² = 0.062). Moreover, we observed a significant effect for transdifferentiation rates across groups (F_3,28_ = 5.83, p=0.003, η² = 0.347), with *post-hoc* analysis showing significantly increased transdifferentiation rates in MD-INF cultures compared to HC, MD and SZ (p=0.004, p=0.009, p=0.026 respectively). Group x treatment interaction was not significantly different (F_3,28_ = 0.52, p=0.669).

### 3.2 Increased IL-6 levels in response to inflammatory stimuli in all subjects

To assess if MDNCs exhibited varying levels of acute inflammation, we performed ELISA for IL-6 on cell supernatants and compared control conditions with LPS treatment (**Figure 4B**). We did not see a significant difference between groups (H(3)=1.59, p=0.661, ϵ²=0.044). However, we found a significant main effect of treatment (LPS vs. control), with IL-6 levels markedly increased in all groups following LPS exposure (H(1)=26.43, p<0.001, ϵ²=0.734), as well as for the interaction of group x treatment (H(7)=29.212, p<0.001, ϵ²=0.811). In Dunn’s *Post Hoc* comparison, IL-6 in MD-INF cells without LPS treatment was significantly lower than in MD-INF cells treated with LPS (p_holm_=0.049).

### 3.3 Heightened neuritic growth in MDNCs of MD patients with elevated inflammatory markers

**Figure 5:**
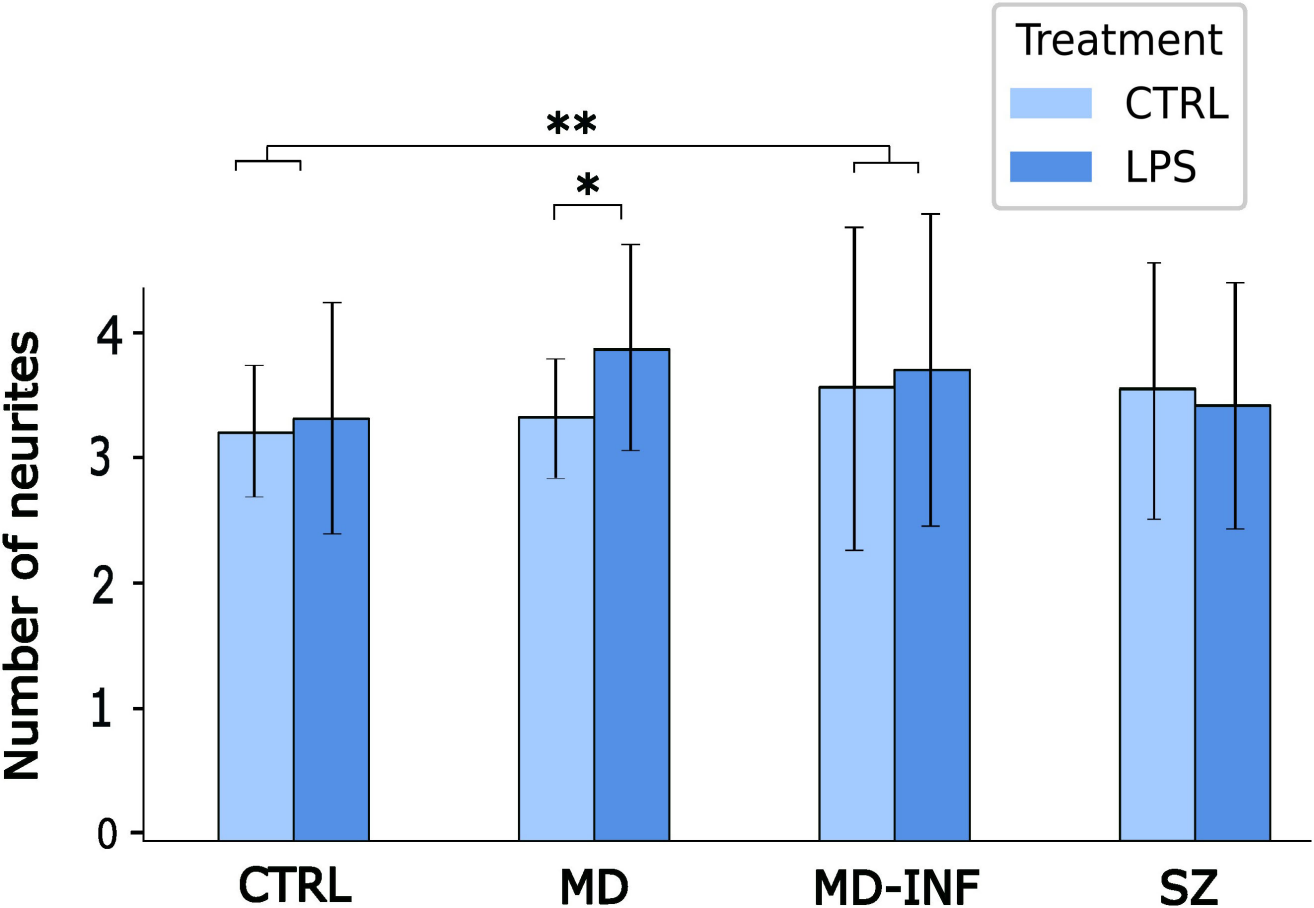
Number of neurites in MDNCs. Cells were pre-selected for having at least three neurites. Analysis showed a significant increase in neurite number in MD-INF compared to HC (p_holm_=0.002). It also revealed a significant increase in neurites in MDNCs from MD patients after LPS treatment compared to untreated MD cells (p_holm_=0.015). Abbreviations: MDNC, monocyte-derived neuronal-like cell; CTRL, control; LPS, lipopolysaccharide; HC, healthy control; MD, patients with major depression; MD-INF, MD patients with elevated inflammation, SZ, schizophrenia patients. Data are displayed as mean ± standard deviation. For statistical analysis, Kruskal-Wallis test was used followed by Dunn’s *post-hoc* test with Holm correction. Statistically significant differences from are indicated by asterisks: **p<0.01 *p<0.05.

To analyze transdifferentiated cells for neuritic growth we applied the same pre-selection criteria used for assessing transdifferentiation rates. Only cells with a minimum of three neurites each exceeding 12 µm in length and less than 5 μm in width were included. We also limited the selection to cells with a soma size of less than 50 μm at its widest point. We then traced and quantified the neurites of these cells. The number of neurites was significantly increased after LPS treatment (H(1)=6.84, p=0.009, ϵ²=0.006). We also found a significant effect of group H(3)=12.87, p=0.005, ϵ²=0.011) and group x treatment interaction (H(7)=31.28, p<0.001, ϵ²=0.027). *Post-hoc* analysis using Dunn’s test showed a significant increase in neurite number in MD-INF compared to HC (p_holm_=0.002) and in MD cells after LPS treatment compared to untreated MD cells (p_holm_=0.015). We observed no other relevant interaction differences (see **Figure 5**).

**Figure 6:**
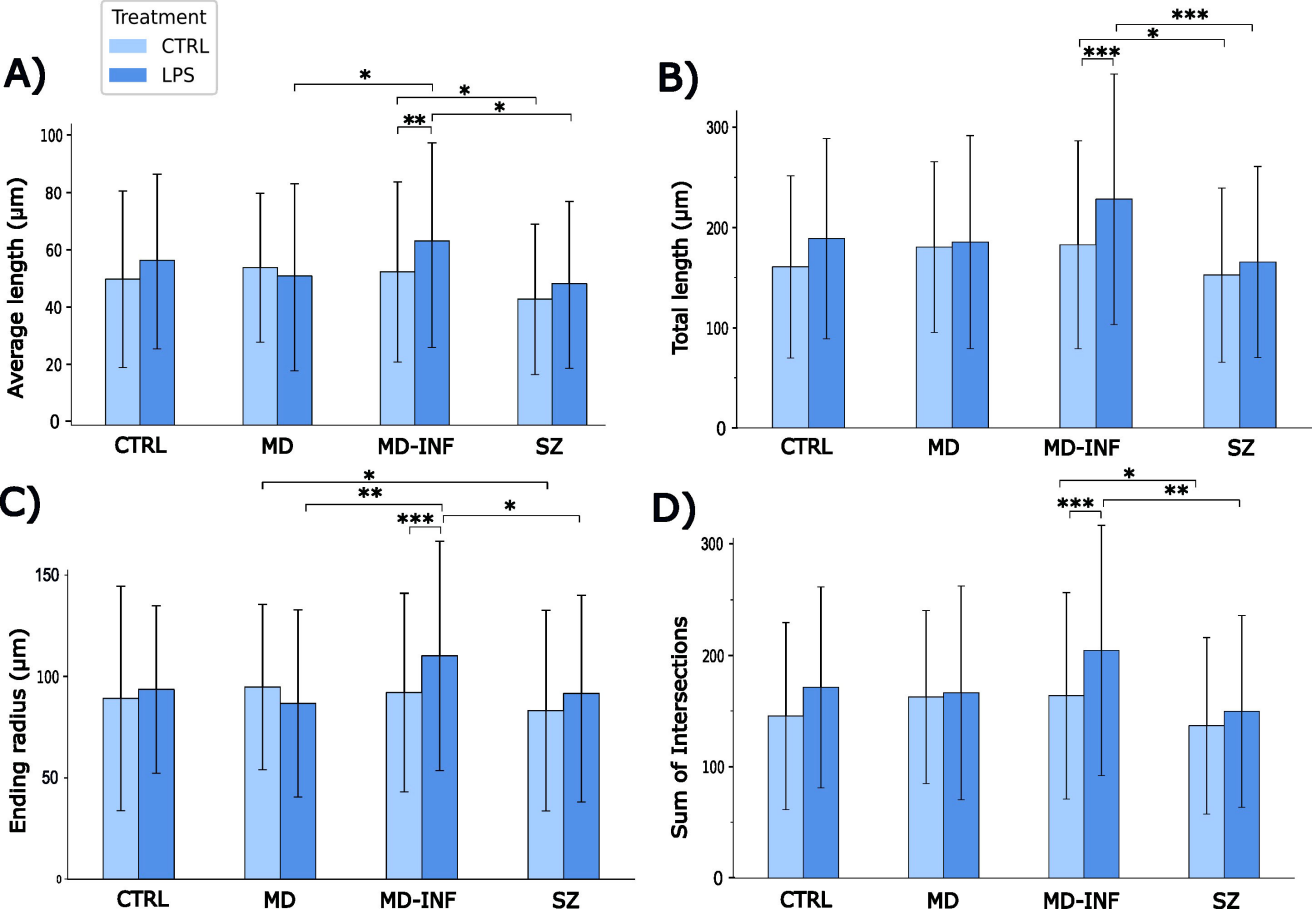
Morphological properties of MDNC neurites. Morphological analysis revealed that neurites of MD-INF cells treated with LPS were longer than those of MD cells treated with LPS (p_holm_=0.021). MD-INF neurites after LPS treatment were longer than MD-INF neurites under control conditions (p_holm_=0.005). Neurites of SZ cells without and with LPS treatment were shorter than neurites of MD-INF cells (p_holm_=0.021 and 0.018 respectively) **(A)**. Neurites of MD-INF cells not treated with LPS were shorter than those of MD-INF cells treated with LPS (p_holm_<0.001). Moreover, neurites of SZ cells were shorter than neurites of MD-INF cells, both without LPS treatment (p_holm_=0.041) and after LPS treatment (p_holm_<0.001) **(B)**. We showed a shorter ending radius for MD-INF cells under control conditions compared to MD-INF cells treated with LPS (p_holm_<0.001). We also found a significantly shorter ending radius for MD cells and SZ cells treated with LPS compared to MD-INF cells treated with LPS (p_holm_=0.002 and 0.012, respectively). It also showed a shorter ending radius for SZ compared to MD neurites without LPS treatment (p_holm_=0.048) **(C)**. MD-INF cells not treated with LPS had a significantly smaller number of intersections compared to MD-INF cells treated with LPS (p_holm_<0.001). In addition, treated and untreated SZ cells had significantly fewer intersections compared to MD-INF cells (p_holm_=0.002 and 0.041, respectively) **(D)**. Abbreviations: MDNC, monocyte-derived neuronal-like cell; CTRL, control; LPS, lipopolysaccharide; HC, healthy control; MD, patients with major depression; MD-INF, MD patients with elevated inflammation, SZ, schizophrenia patients. Data are displayed as mean ± standard deviation. For statistical analyses Kruskal-Wallis test was used followed by Dunn’s *post-hoc* test with Holm correction. Statistically significant differences are indicated by asterisks: *p<0.05, **p<0.01, ***p<0.001.

The average neurite length was significantly affected by group (H(3)=23.5, p<0.001, ϵ²=0.02) and increased by LPS treatment (H(1)=11.39, p<0.001, ϵ²=0.01). Group x treatment interaction also reached significance (H(7)=45.21, p<0.001; ϵ²=0.039, **Figure 6A**). *Post-hoc* analysis indicated that neurites of MD-INF cells treated with LPS were longer than those of MD cells treated with LPS (p_holm_ =0.021). MD-INF neurites after LPS treatment were longer than MD-INF neurites under control conditions (p_holm_ =0.005). Neurites of SZ cells without and with LPS treatment were shorter than neurites of MD-INF cells (p_holm_=0.021; p_holm_ =0.018 respectively).

The total neurite length was significantly affected by group (H(3)=29.24, p<0.001, ϵ²=0.025) and increased by LPS treatment (H(1)=18.12, p<0.001, ϵ²=0.016). The interaction between group and treatment also reached significance (H(7)=52.38, p<0.001, ϵ²=0.045; **Figure 6B**). *Post-hoc* analysis indicated that neurites of MD-INF cells not treated with LPS were shorter than those of MD-INF cells treated with LPS (p_holm_<0.001). Moreover, neurites of SZ cells were shorter than neurites of MD-INF cells, both without LPS treatment (p_holm_=0.041) and after LPS treatment (p_holm_<0.001). Neurites of MD cells not treated with LPS were not significantly shorter than those of untreated SZ cells but showed a trend toward reduced length (p_holm_=0.072).

To further determine the complexity of the traced neurites, we performed Sholl analysis. The ending radius differed significantly between groups (H(3)=18.62, p<0.001, ϵ²=0.016, **Figure 6C**). We also found a significant group x treatment effect (H(7)=42.67, p<0.001, ϵ²=0.037) and a main effect of LPS treatment (H(1)=10.23, p=0.001, ϵ²=0.009). *Post-hoc* comparison with Dunn’s test showed a shorter ending radius for MD-INF cells under control conditions compared to MD-INF cells treated with LPS (p_holm_ <0.001). We also found a significantly shorter ending radius for MD cells and SZ cells treated with LPS compared to MD-INF cells treated with LPS (p_holm_=0.002; p_holm_=0.012, respectively). It also showed a shorter ending radius for SZ compared to MD neurites without LPS treatment (p_holm_ =0.048).

The sum of Sholl intersections differed significantly across group (H(3)=27.68, p<0.001, ϵ²=0.024) and increased following LPS treatment (H(1)=18.41, p<0.001, ϵ²=0.016). The group x treatment interaction also reached significance (H(7)=50.91, p<0.001, ϵ²=0.044; **Figure 6D**). *Post-hoc* comparison showed that MD-INF cells not treated with LPS had a significantly smaller number of intersections compared to MD-INF cells treated with LPS (p_holm_<0.001). In addition, treated and untreated SZ cells had significantly fewer intersections compared to MD-INF cells (p_holm_=0.002; p_holm_=0.041, respectively). Under control conditions, SZ cells showed a trend toward fewer intersections compared to MD cells (p_holm_=0.064).

## 4 Discussion

As of today, the complex relationship between inflammation and depression at a cellular level remains inadequately understood. Increasing evidence shows an association between elevated inflammatory markers in both serum and cerebrospinal fluid, severity of depression (22), and treatment resistance (7). Increased peripheral inflammation appears to be linked to neuroinflammation, potentially through mechanisms such as elevated blood-brain-barrier permeability (8). Neuroinflammation has been observed to correlate with a decline in hippocampal neurogenesis (23). Furthermore, there is evidence suggesting a potential association between inflammation and smaller hippocampal volume (24), similar to findings in patients with depression (25). Evidence also suggests that neuroinflammation is involved in the regulation of adult neurogenesis and neuritogenesis in the hippocampus (26). In addition, increased peripheral CRP levels have been linked with decreased functional connectivity of the striatum and the ventromedial frontal cortex, which was associated with anhedonia and psychomotor slowing (27).

Our findings show higher transdifferentiation rates in MDNCs from MD-INF patients under baseline conditions. Following LPS stimulation, transdifferentiation rates increased in MDNCs from all groups. While neuritic growth was unaffected under baseline conditions, LPS stimulation led to a significant increase across all groups. MDNCs from MD-INF patients were particularly responsive to LPS treatment, showing significantly higher arborization and total length of neurites compared to all other patient groups. These results suggest hyperresponsiveness to inflammation, especially among individuals with depression related to chronic inflammation. Here, we define ‘hyperresponsiveness’ as an increased sensitivity of MDNCs to acute inflammatory stimulation, reflected by enhanced neurite outgrowth and higher transdifferentiation rates compared to other groups.

In line with our findings, prior research suggests that inflammation is not always detrimental. When examining the impact on neural stem cells, inflammation shows dichotomous effects, either supporting or inhibiting proliferation, survival and differentiation (28). Chronic inflammation seems to impair the function of brain stem cells (29), reduces the dendritic length of immature new-born granule cells in the hippocampus of rodents (10), and leads to a dysregulation of hippocampal neurogenesis (23). However, emerging evidence highlights the constructive role of acute inflammation in promoting regenerative capacities in different tissues (28). For example, in the adult zebrafish brain, acute inflammation triggers reactive proliferation of neural stem and progenitor cells (30). In adult mice, inflammatory stimuli increase hippocampal neural precursor cell proliferation (31). In adult rats, ischemic stroke - an acute inflammatory event – induces increased cell division in the subventricular zone, possibly indicating enhanced neuronal differentiation and adult neurogenesis (32). Compensatory neurogenesis after ischemic stroke has also been observed in the human brain (33). Physical exercise, which is a well-known promoter of adult neurogenesis in the hippocampus (34), also (temporarily) elevates peripheral inflammatory markers (35). Together, these findings support the notion that inflammation can act as a critical factor in neurogenesis (36–38). Thus, whether inflammation proves beneficial or detrimental may partly depend on the intensity and the duration of the inflammatory stimulus. *In vitro* experiments have shown that high concentrations of corticosterone induce cell death in neuronal precursor cells, whereas low concentrations promote proliferation in the hippocampus of adult mice (31).

In our study, MDNCs of patients with depression and preexisting elevated inflammatory markers showed higher transdifferentiation rates and longer dendrites after an acute inflammatory stimulus than patients without peripheral inflammation or healthy controls. This finding may suggest a protective regulatory mechanism by enhancing neurogenesis, cell growth or differentiation specifically in the subgroup of depressed patients with preexisting inflammation. An alternative interpretation may be that increased growth and differentiation in the cells of this patient group may mark the onset of a dysregulated immunoregulatory response characterized by aberrant cellular growth, eventually leading to cell death. Our LPS stimulation lasted 24 h, which can be considered a short-term stimulus. As mentioned, acute inflammatory impulses might have a beneficial effect on cell proliferation. The reaction of MDNCs to long-term LPS stimulation remains to be investigated.

Even in network neuroscience, a common finding is that damaging a neuronal network may result in a regional increase in functional connectivity. This unexpected increase in neural connection is also referred to as hyperconnectivity (39). Short-term hyperconnectivity appears to be adaptive but chronic hyperconnectivity may eventually lead to pathological hypoconnectivity (39) as found especially in TRD (27, 40, 41). In our study, neuronal-like cells from patients with depression and inflammation displayed higher sensitivity to inflammatory stimuli resulting in increased neuritic growth. Increased neuritic growth may initially lead to hyperconnectivity, which could later transition to hypoconnectivity, potentially contributing to the chronicity of depression.

A notable limitation of our study is that we did not measure the influence of psychopharmacological treatment. Each participant received different antidepressant (MD, MD-INF) or antipsychotic (SZ) medications. Antidepressants have been shown to increase differentiation and proliferation (42), which could have confounded our findings. Another constraint lies in the absence of microglia in our culture, which are known to play a vital role in the regulation of neuroinflammation (43). The presence of microglia in our culture could have influenced the outcomes.

When recruiting participants, we did not control for gender, as we did not expect gender to influence the outcome of our cell model research. By chance, the HC and MD groups included only female participants whereas the MD-INF and SZ group included only males. Although the sample size in each group was relatively small and this gender imbalance may represent a potential confounding factor, it is unlikely to account for the observed differences across groups or the relatively consistent within-group measures. Gender differences at the organismal level are primarily mediated by endocrine factors, which are absent under ex vivo cell culture conditions. Nonetheless, differences in immune or cellular responses between male and female derived samples cannot be entirely excluded. Future studies should aim to replicate these findings in gender-balanced cohorts to further validate the robustness of the observed effects.

Additionally, the use of different pharmacological treatments among participants represents a potential confounding factor, although it reflects the clinical heterogeneity typically observed in real-world patient populations.

Another limitation of our study design is that it does not allow us to definitively attribute the observed increase in IL-6 levels following inflammatory stimulation to MDNCs, as undifferentiated monocytes were also present in the culture. Future studies should aim to clarify the cellular source of IL-6 under these conditions.

Baseline neurite measurements were recorded for all groups, allowing assessment of intrinsic differences in differentiation and neurite growth prior to any acute inflammatory stimulus. While experiments were not blinded to group, all cultures were processed under standardized conditions to reduce potential procedural bias. This supports the interpretation that observed effects following LPS mainly reflect cellular responses to the stimulus rather than pre-existing variability or technical differences. Nevertheless, the absence of blinding may introduce a degree of observer bias, particularly in the manual steps of cell selection and neurite tracing. Future studies could further reduce this risk by implementing blinded analyses, for instance through coded sample labeling.

We note that the number of transdifferentiated cells differed between participants, resulting in unequal numbers of cell-level measurements across groups. Future studies with larger sample sizes will be valuable to confirm these findings with fully participant-level analyses.

In conclusion, our findings suggest a hyperresponsiveness of MDNCs in patients with depression and elevated inflammatory markers to acute inflammatory stimuli, possibly leading to higher differentiation rates and heightened neuritic growth. Neuroinflammation appears to be intricately regulated and demonstrates bidirectional changes. The cellular response to inflammation depends on factors such as the timing of inflammation onset as well as the intensity and duration of the stimulus. The MDNC model may provide valuable insights into the complex regulatory mechanisms and interindividual variability in inflammatory responses among patients with depression.

## Data Availability

The raw data supporting the conclusions of this article will be made available by the authors, without undue reservation.

## References

[1] Disease GBD Injury I Prevalence C. Global, regional, and national incidence, prevalence, and years lived with disability for 354 diseases and injuries for 195 countries and territories, 1990-2017: a systematic analysis for the Global Burden of Disease Study 2017. Lancet. (2018) 392:1789–858. doi: 10.1016/S0140-6736(18)32279-7, PMID: 30496104 PMC6227754

[2] Friedrich MJ. Depression is the leading cause of disability around the world. JAMA. (2017) 317:1517. doi: 10.1001/jama.2017.3826, PMID: 28418490

[3] Touloumis C. The burden and the challenge of treatment-resistant depression. Psychiatriki. (2021) 32:11–4. doi: 10.22365/jpsych.2021.046, PMID: 34990376

[4] Reif A Bitter I Buyze J Cebulla K Frey R Fu DJ. Esketamine nasal spray versus quetiapine for treatment-resistant depression. N Engl J Med. (2023) 389:1298–309. doi: 10.1056/NEJMoa2304145, PMID: 37792613

[5] Beurel E Toups M Nemeroff CB. The bidirectional relationship of depression and inflammation: double trouble. Neuron. (2020) 107:234–56. doi: 10.1016/j.neuron.2020.06.002, PMID: 32553197 PMC7381373

[6] Suneson K Grudet C Ventorp F Malm J Asp M Westrin A. An inflamed subtype of difficult-to-treat depression. Prog Neuropsychopharmacol Biol Psychiatry. (2023) 125:110763. doi: 10.1016/j.pnpbp.2023.110763, PMID: 37037323

[7] Strawbridge R Arnone D Danese A Papadopoulos A Herane Vives A Cleare AJ. Inflammation and clinical response to treatment in depression: A meta-analysis. Eur Neuropsychopharmacol. (2015) 25:1532–43. doi: 10.1016/j.euroneuro.2015.06.007, PMID: 26169573

[8] Hassamal S. Chronic stress, neuroinflammation, and depression: an overview of pathophysiological mechanisms and emerging anti-inflammatories. Front Psychiatry. (2023) 14:1130989. doi: 10.3389/fpsyt.2023.1130989, PMID: 37252156 PMC10213648

[9] Troubat R Barone P Leman S Desmidt T Cressant A Atanasova B. Neuroinflammation and depression: A review. Eur J Neurosci. (2021) 53:151–71. doi: 10.1111/ejn.14720, PMID: 32150310

[10] Llorens-Martin M Jurado-Arjona J Fuster-Matanzo A Hernandez F Rabano A Avila J. Peripherally triggered and GSK-3beta-driven brain inflammation differentially skew adult hippocampal neurogenesis, behavioral pattern separation and microglial activation in response to ibuprofen. Transl Psychiatry. (2014) 4:e463. doi: 10.1038/tp.2014.92, PMID: 25313506 PMC4350524

[11] Tartt AN Mariani MB Hen R Mann JJ Boldrini M. Dysregulation of adult hippocampal neuroplasticity in major depression: pathogenesis and therapeutic implications. Mol Psychiatry. (2022) 27:2689–99. doi: 10.1038/s41380-022-01520-y, PMID: 35354926 PMC9167750

[12] Fond G Lancon C Korchia T Auquier P Boyer L. The role of inflammation in the treatment of schizophrenia. Front Psychiatry. (2020) 11:160. doi: 10.3389/fpsyt.2020.00160, PMID: 32256401 PMC7093323

[13] Takahashi K Yamanaka S. Induction of pluripotent stem cells from mouse embryonic and adult fibroblast cultures by defined factors. Cell. (2006) 126:663–76. doi: 10.1016/j.cell.2006.07.024, PMID: 16904174

[14] Urbach A Bar-Nur O Daley GQ Benvenisty N. Differential modeling of fragile X syndrome by human embryonic stem cells and induced pluripotent stem cells. Cell Stem Cell. (2010) 6:407–11. doi: 10.1016/j.stem.2010.04.005, PMID: 20452313 PMC3354574

[15] Pera MF. Stem cells: The dark side of induced pluripotency. Nature. (2011) 471:46–7. doi: 10.1038/471046a, PMID: 21368819

[16] Bellon A Wegener A Lescallette AR Valente M Yang S-K Gardette R. Transdifferentiation of human circulating monocytes into neuronal-like cells in 20 days and without reprograming. Front Mol Neurosci. (2018) 11. doi: 10.3389/fnmol.2018.00323, PMID: 30760979 PMC6156467

[17] Zubin J Spring B. Vulnerability–a new view of schizophrenia. J Abnorm Psychol. (1977) 86:103–26. doi: 10.1037/0021-843X.86.2.103 858828

[18] Sheehan DV Lecrubier Y Sheehan KH Amorim P Janavs J Weiller E. The Mini-International Neuropsychiatric Interview (M.I.N.I.): the development and validation of a structured diagnostic psychiatric interview for DSM-IV and ICD-10. J Clin Psychiatry. ((1998)) 59:22–33., PMID: 9881538

[19] Arshadi C Gunther U Eddison M Harrington KIS Ferreira TA. SNT: a unifying toolbox for quantification of neuronal anatomy. Nat Methods. (2021) 18:374–7. doi: 10.1038/s41592-021-01105-7, PMID: 33795878

[20] Schindelin J Arganda-Carreras I Frise E Kaynig V Longair M Pietzsch T. Fiji: an open-source platform for biological-image analysis. Nat Methods. (2012) 9:676–82. doi: 10.1038/nmeth.2019, PMID: 22743772 PMC3855844

[21] Ferreira TA Blackman AV Oyrer J Jayabal S Chung AJ Watt AJ. Neuronal morphometry directly from bitmap images. Nat Methods. (2014) 11:982–4. doi: 10.1038/nmeth.3125, PMID: 25264773 PMC5271921

[22] Osimo EF Baxter LJ Lewis G Jones PB Khandaker GM. Prevalence of low-grade inflammation in depression: a systematic review and meta-analysis of CRP levels. Psychol Med. (2019) 49:1958–70. doi: 10.1017/S0033291719001454, PMID: 31258105 PMC6712955

[23] Wu A Zhang J. Neuroinflammation, memory, and depression: new approaches to hippocampal neurogenesis. J Neuroinflammation. (2023) 20:283. doi: 10.1186/s12974-023-02964-x, PMID: 38012702 PMC10683283

[24] Frodl T Carballedo A Hughes MM Saleh K Fagan A Skokauskas N. Reduced expression of glucocorticoid-inducible genes GILZ and SGK-1: high IL-6 levels are associated with reduced hippocampal volumes in major depressive disorder. Transl Psychiatry. (2012) 2:e88. doi: 10.1038/tp.2012.14, PMID: 22832853 PMC3309536

[25] Videbech P Ravnkilde B. Hippocampal volume and depression: a meta-analysis of MRI studies. Am J Psychiatry. (2004) 161:1957–66. doi: 10.1176/appi.ajp.161.11.1957, PMID: 15514393

[26] Amanollahi M Jameie M Heidari A Rezaei N. The dialogue between neuroinflammation and adult neurogenesis: mechanisms involved and alterations in neurological diseases. Mol Neurobiol. (2023) 60:923–59. doi: 10.1007/s12035-022-03102-z, PMID: 36383328

[27] Felger JC Li Z Haroon E Woolwine BJ Jung MY Hu X. Inflammation is associated with decreased functional connectivity within corticostriatal reward circuitry in depression. Mol Psychiatry. (2016) 21:1358–65. doi: 10.1038/mp.2015.168, PMID: 26552591 PMC4862934

[28] Kizil C Kyritsis N Brand M. Effects of inflammation on stem cells: together they strive? EMBO Rep. (2015) 16:416–26. doi: 10.15252/embr.201439702, PMID: 25739812 PMC4388609

[29] Pluchino S Muzio L Imitola J Deleidi M Alfaro-Cervello C Salani G. Persistent inflammation alters the function of the endogenous brain stem cell compartment. Brain. (2008) 131:2564–78. doi: 10.1093/brain/awn198, PMID: 18757884 PMC2570715

[30] Kyritsis N Kizil C Zocher S Kroehne V Kaslin J Freudenreich D. Acute inflammation initiates the regenerative response in the adult zebrafish brain. Science. (2012) 338:1353–6. doi: 10.1126/science.1228773, PMID: 23138980

[31] Wolf SA Steiner B Wengner A Lipp M Kammertoens T Kempermann G. Adaptive peripheral immune response increases proliferation of neural precursor cells in the adult hippocampus. FASEB J. (2009) 23:3121–8. doi: 10.1096/fj.08-113944, PMID: 19433626

[32] Zhang R Zhang Z Zhang C Zhang L Robin A Wang Y. Stroke transiently increases subventricular zone cell division from asymmetric to symmetric and increases neuronal differentiation in the adult rat. J Neurosci. (2004) 24:5810–5. doi: 10.1523/JNEUROSCI.1109-04.2004, PMID: 15215303 PMC6729213

[33] Ceanga M Dahab M Witte OW Keiner S. Adult neurogenesis and stroke: A tale of two neurogenic niches. Front Neurosci. (2021) 15:700297. doi: 10.3389/fnins.2021.700297, PMID: 34447293 PMC8382802

[34] Gao Y Syed M Zhao X. Mechanisms underlying the effect of voluntary running on adult hippocampal neurogenesis. Hippocampus. (2023) 33:373–90. doi: 10.1002/hipo.23520, PMID: 36892196 PMC10566571

[35] Stranahan AM Lee K Mattson MP. Central mechanisms of HPA axis regulation by voluntary exercise. Neuromolecular Med. (2008) 10:118–27. doi: 10.1007/s12017-008-8027-0, PMID: 18273712 PMC3010733

[36] Rahman AA Amruta N Pinteaux E Bix GJ. Neurogenesis after stroke: A therapeutic perspective. Transl Stroke Res. (2021) 12:1–14. doi: 10.1007/s12975-020-00841-w, PMID: 32862401 PMC7803692

[37] Dillen Y Kemps H Gervois P Wolfs E Bronckaers A. Adult neurogenesis in the subventricular zone and its regulation after ischemic stroke: implications for therapeutic approaches. Transl Stroke Res. (2020) 11:60–79. doi: 10.1007/s12975-019-00717-8, PMID: 31309427

[38] Jin K Wang X Xie L Mao XO Zhu W Wang Y. Evidence for stroke-induced neurogenesis in the human brain. Proc Natl Acad Sci U S A. (2006) 103:13198–202. doi: 10.1073/pnas.0603512103, PMID: 16924107 PMC1559776

[39] Hillary FG Grafman JH. Injured brains and adaptive networks: the benefits and costs of hyperconnectivity. Trends Cognit Sci. (2017) 21:385–401. doi: 10.1016/j.tics.2017.03.003, PMID: 28372878 PMC6664441

[40] Ge R Torres I Brown JJ Gregory E McLellan E Downar JH. Functional disconnectivity of the hippocampal network and neural correlates of memory impairment in treatment-resistant depression. J Affect Disord. (2019) 253:248–56. doi: 10.1016/j.jad.2019.04.096, PMID: 31060011

[41] Abdallah CG Averill CL Salas R Averill LA Baldwin PR Krystal JH. Prefrontal connectivity and glutamate transmission: relevance to depression pathophysiology and ketamine treatment. Biol Psychiatry Cognit Neurosci Neuroimaging. (2017) 2:566–74. doi: 10.1016/j.bpsc.2017.04.006, PMID: 29034354 PMC5635826

[42] Ikhsan M Palumbo A Rose D Zille M Boltze J. Neuronal stem cell and drug interactions: A systematic review and meta-analysis: concise review. Stem Cells Transl Med. (2019) 8:1202–11. doi: 10.1002/sctm.19-0020, PMID: 31313515 PMC6811698

[43] Wang H He Y Sun Z Ren S Liu M Wang G. Microglia in depression: an overview of microglia in the pathogenesis and treatment of depression. J Neuroinflammation. (2022) 19:132. doi: 10.1186/s12974-022-02492-0, PMID: 35668399 PMC9168645

